# Barrasford Sanatorium's Finances

**Published:** 1919-04-05

**Authors:** Cecil G. R. Goodwin

**Affiliations:** Medical Superintendent. Neil Brodie, Secretary. The Sanatorium, Barrasford, S.O., Northumberland.


*To the Editor of* The Hospital.

### Barrasford Sanatorium's Finances.

*To the Editor of* The Hospital.

Sir,—With reference to your note in the issue of The Hospital of March 22, 1919, concerning the increase in the number of beds at the Barrasford Sanatorium as an attempt to lessen the financial loss at which the sanatorium has been run during the past year, we thank you for your approval of the measure which the Committee have taken, and for your good wishes. With regard to the likelihood of the success of the policy, it is difficult to speak with certainty so soon, but the figures have been carefully gone into, and it would appear that if the 85 beds can be kept fully occupied then the expenditure should approximately equal the income at the reduced fee per head, or at any rate any deficit should be comparatively small. The basis of the calculation, however, is that the 85 beds should be kept continuously full. It is encouraging to be able to state that the number of beds now let to various bodies is 64, and over and above this number there are some private cases in residence.

The Barrasford Sanatorium though exceptionally well placed from the point of view of efficient treatment, is very isolated, and the maintenance of the institution is an expensive business, in that we are forced to make our own electric light, pump our own water, to have coal led from a distance, &c. Therefore, as the number of rooms in the sanatorium remains as heretofore, and only the number of beds is augmented, there is necessary only a slight increase in the amount of light and fuel and number of staff to deal with an added number of patients. The assumption is, then, that whereas the amount from fees, if all the beds are filled, will be increased, yet the cost of administration of the institution will be little greater. The point that the increased number of patients will need feeding has not of course been overlooked.

For a very long time it has been stated in the Press that the call for sanatorium beds has far exceeded the number of available beds in the country; it would, therefore, appear likely that our remaining empty beds should soon fill when it is known that they are available at fees which compare favourably with those at other sanatoria of similar status. It will be interesting to observe the result of the Committee's policy, and we are of the opinion that it will be found to be successful.

We are, &c.,

Cecil G. R. Goodwin, Medical Superintendent.
Neil Brodie, Secretary.

The Sanatorium, Barrasford, S.O., Northumberland.
March 25, 1919.

